# Estimated Protection Too Conservative

**DOI:** 10.1371/journal.pmed.0030065

**Published:** 2006-01-31

**Authors:** James D Shelton

**Affiliations:** **1**United States Agency for International DevelopmentWashington, D.CUnited States of America

The randomized study by Auvert et al. [ 1] on male circumcision to prevent HIV infection is clearly a landmark study, which supports compelling observational evidence of strong protection [ 2]. However, I believe the highlighted 60% degree of protection from the “intent-to-treat” analysis is probably too conservative. Deep in the article, we learn that the “per protocol” analysis, which addressed the 10.3% of men allocated to the noncircumcision group who nevertheless decided to be circumcised outside of the study, found a relative risk of 0.24%—or a protective effect of 76%.

Some might argue that the intent-to-treat analysis is more scientific, and reduces the impact of some selection or behavioral bias in those who opted for circumcision notwithstanding their allocation to the control arm. For example, it could be that men allocated to the noncircumcision group who were predisposed to less risky behavior but wanted to be extra safe might have chosen to be circumcised. In the opposite direction, those who were indulging most in risky behavior might have chosen to be circumcised to reduce their risk.

In my view, however, if we are interested in the true biologic effect, it is not very scientific to bias in favor of the analysis with a 10% contamination of that biological effect. In this study, we are fortunate enough to have quite richly reported behavioral data. More analysis of the per protocol analysis should have been presented, including the behavior of the crossovers. It is reasonably likely that any difference in the behavior of the crossovers would have little impact since, in the intent-to-treat analysis, adjustment for the increase in riskier behavior in the treatment group had little effect on the overall result.

The paper should have had prominent presentation of both analyses. In either case, the protection is quite substantial. But from an epidemiologic and personal perspective, what amounts to a failure rate of 40% versus 24% could be quite important.
